# Prevalence and Co-Occurrence of Psychiatric Conditions Among Bereaved Adults

**DOI:** 10.1001/jamanetworkopen.2024.15325

**Published:** 2024-06-06

**Authors:** Alyssa A. Rheingold, Joah L. Williams, Jamison S. Bottomley

**Affiliations:** 1 National Crime Victims Research and Treatment Center, Department of Psychiatry and Behavioral Sciences, Medical University of South Carolina, Charleston; 2 Department of Psychology, University of Memphis, Memphis, Tennessee

## Abstract

**Importance:**

Rates of grief-related psychiatric conditions, such as prolonged grief disorder (PGD), posttraumatic stress disorder (PTSD), and major depressive disorder (MDD), among bereaved adults in the US are largely unknown due to limited studies that leverage national samples.

**Objective:**

To assess risk factors for and prevalence rates and co-occurrence of PGD, PTSD, and MDD among bereaved adults in the US.

**Design, Setting, and Participants:**

Data for this survey study were collected from a large US panel sample between October 10 and 28, 2022, using a web-based survey. Participants were aged 18 years or older and were proficient in English. Data analysis was conducted between March and June 2023.

**Main Outcomes and Measures:**

Probable psychiatric conditions were assessed with validated measures that used conservative cutoff scores, duration, and impairment criteria. These measures included the revised Prolonged Grief Disorder scale for PGD, the PTSD Checklist for *DSM-5* for PTSD, and the Patient Health Questionnaire-9 for MDD. Data were analyzed using basic descriptives and logistic regression.

**Results:**

A total of 2034 adults (n = 1529 and 505 in the bereaved and comparison groups, respectively) completed the study. Respondents had a mean (SD) age of 40.7 (15.9) years; the majority were women (1314 [64.6%]) and had at least some college experience (1394 [68.5%]). With regard to race and ethnicity, 392 respondents (19.3%) were Black, 138 (6.8%) were Hispanic, and 1357 (66.7%) were White. Among bereaved adults, 312 (20.4%), 518 (33.9%), and 461 (30.2%) met criteria for a presumptive diagnosis of PGD, PTSD, and MDD, respectively. Comorbidities were common, with 441 participants (28.8%) meeting criteria for at least 2 co-occurring disorders. Comorbid PGD, PTSD, and MDD were more common than any 2 co-occurring or isolated disorders; the presence of co-occurring conditions was more likely among respondents who reported a traumatic loss. Age and educational attainment were associated with the risk of psychiatric conditions; less time since the index death, loss of a psychologically close other, and a traumatic loss experience were associated with increased risk of PGD, PTSD, and MDD or their co-occurrence.

**Conclusions and Relevance:**

In this study, the majority of bereaved adults did not meet presumptive criteria for PGD, PTSD, or MDD. Nevertheless, PGD, PTSD, and MDD were highly prevalent and comorbid, particularly among those who experienced traumatic loss. These findings underscore the need for integrated psychological care that leverages transdiagnostic mechanisms of evidence-based practice.

## Key Points

**Question:**

What are the risk factors for and prevalence rates and co-occurrence of grief-related psychiatric conditions among bereaved adults in the US?

**Findings:**

In this survey study of 2034 adults, presumptive prevalence rates of 20% for prolonged grief disorder (PGD), 34% for posttraumatic stress disorder (PTSD), and 30% for major depressive disorder (MDD) were observed among 1529 bereaved respondents. Comorbidities were also common, as reported by 29% of respondents; the risk of grief-related psychiatric conditions was greatest among those who experienced the traumatic loss of a psychologically close other.

**Meaning:**

Given that loss is a risk factor for the co-occurrence of PGD, PTSD, and MDD, these findings suggest that transdiagnostic treatment may be most efficient and effective.

## Teaser

This survey study evaluates risk factors for and prevalence rates and co-occurrence of grief-related psychiatric conditions among bereaved adults in the US.

## Introduction

Grief in response to the death of a loved one is a nearly universal experience. For the majority of bereaved persons, acute grief is typically accompanied by moderate disruptions in psychological, physical, and interpersonal functioning and generally resolves within 1 year.^1^ A minority of bereaved individuals, however, continue to experience chronic grief symptoms and impairment long after loss, leading to the recognition of prolonged grief disorder (PGD) in the *Diagnostic and Statistical Manual of Mental Disorders, Fifth Edition, Text Revision* (*DSM-5-TR*).^2^ Symptoms of PGD include persistent grief characterized by yearning for or preoccupation with the decedent, accompanied by at least 3 of 8 additional mood or distress symptoms.^2^ Despite some symptom overlap with other psychiatric conditions,^3^ PGD has a distinct etiology compared with posttraumatic stress disorder (PTSD) and depression.^4^ Whereas PTSD is characterized by traumatic distress after a traumatic event,^5^ PGD is chiefly characterized by separation distress after interpersonal loss through death.^6^ Although bereavement can precipitate a depressive episode, depression is largely theorized to occur as a result of deficient response-contingent positive reinforcement.^7^

Prior research suggests that individuals may be at risk of mental health difficulties based on the type of loss experienced. For instance, studies with subpopulations of loss survivors indicate that those who experience a traumatic loss are at risk of PGD, PTSD, and depressive symptomatology,^8^ which often co-occur and are highly prevalent in this population.^3,9,10^ (For the purposes of this study, we define *traumatic loss* as a death that involves violence or human volition.) The purported prevalence rate of PGD is approximately 49% among traumatic loss survivors^11^ compared with approximately 9.8% among individuals bereaved by a natural loss.^12^ Studies investigating mood-related symptoms among traumatic loss survivors have reported high levels of depression^13^ and increased suicide risk, particularly among suicide and overdose loss survivors.^9,14^

Despite some initial findings of risk for chronic mental health difficulties among specific subpopulations of traumatic loss survivors (eg, due to homicide, motor vehicle crash, drug-related overdose, or suicide), no studies to date using US samples of bereaved individuals have examined PGD, PTSD, and major depressive disorder (MDD) comparing the nature of the death. Therefore, this study aimed to examine risk factors for and prevalence rates and co-occurrence of PGD, PTSD, and MDD among a large, national, convenience sample of US adults bereaved by traumatic and natural losses compared with those without a history of loss.

## Methods

All procedures involved in this survey study were reviewed and approved by the Medical University of South Carolina Institutional Review Board. Recruitment and sampling procedures are presented in the eFigure in Supplement 1. Participants provided informed consent upon survey initiation. The study followed the Strengthening the Reporting of Observational Studies in Epidemiology (STROBE) reporting guideline for cross-sectional studies and the American Association for Public Opinion Research (AAPOR) guideline for survey studies.

### Data Collection and Sample

Data collection occurred between October 10 and 28, 2022, through a web-based panel survey. The survey was distributed by Qualtrics Panel Services to gather information from a large number of US adults (aged ≥18 years) who were proficient in English and who (1) had not experienced the death of a loved one (eg, family member or close friend) in their lifetime, (2) had experienced the death of a loved one due to natural causes (eg, cancer, Alzheimer disease, heart attack, or stroke), or (3) had experienced the death of a loved one due to a traumatic loss (eg, homicide, motor vehicle crash, drug-related overdose, or suicide). Qualtrics Panel Services partners with more than 20 online panel providers to generate a large pool of study respondents for psychological research,^15^ including in the areas of traumatic stress^16^ and bereavement,^17^ producing data that closely approximate representative samples relative to other online survey panel platforms.^18,19^ Incentives for survey completion included money, gift cards, airline miles, and vouchers and were based on panelist preference and complexity of recruitment.

Recruitment occurred online via Qualtrics Panel Services. To screen participants and achieve a priori sample sizes (ie, survey quotas) for the 3 aforementioned subgroups, eligible participants responded to a series of questions pertaining to their lifetime experiences of bereavement. Prespecified sample sizes for each subgroup were determined through a series of power analyses. Once a priori sample sizes were met for each subgroup, data collection for that subgroup was concluded.

To ensure the validity of survey responses, Qualtrics used several strategies. These strategies included the use of deduplication technology to eliminate redundant responses, use of digital fingerprint technology to confirm that internet provider addresses were used only once, and elimination of invalid responses (eg, those completed in less than one-third of the average completion time).

### Survey Measures

Respondents completed online surveys using branching-format questions that assessed a variety of experiences and mental health symptoms. Demographic information (eg, age, race and ethnicity, and gender identity) was collected via self-report to examine potential disparities in mental health outcomes. Race and ethnicity were reported as Black, Hispanic, White, or other race or ethnicity (which comprised American Indian or Alaska Native, Asian, Native Hawaiian or Other Pacific Islander, or multiple races or ethnicities). Due to a low response rate of individuals identifying as nonbinary, gender was categorized in a dichotomous fashion (male or female) in all analyses.

Predeath psychological closeness to the decedent was assessed using a sliding scale with values ranging from 1 to 10. Other loss-related factors were assessed, such as decedent kinship category and time since death (captured initially in months and later categorized in years for ease of interpretation).

Prolonged grief disorder was assessed using the revised version of the Prolonged Grief Disorder scale (PG-13-R).^20^ The PG-13-R is a self-report instrument that assesses PGD symptoms in accordance with *DSM-5-TR* criteria.^2^ Scores of 30 or greater in combination with a positive response on the impairment criterion indicate the probable presence of PGD.^20^ Posttraumatic stress disorder was assessed using the PTSD Checklist for *DSM-5* (PCL-5).^21^ Respondents were asked to respond to all 20 items with the traumatic loss or natural death of a loved one as the index event. For those reporting no history of loss experiences, the PCL-5 was anchored to a past traumatic experience, if applicable. Finally, MDD was assessed using the Patient Health Questionnaire-9,^22^ a 9-item self-report measure. For this study, the recommended cutoff score of 10 was used to identify individuals with positive screening results for MDD.^23^

### Statistical Analysis

Statistical analyses occurred in 3 stages. First, we calculated demographic and loss-related characteristics of the sample and assessed for statistically significant differences among these variables across study subgroups (ie, no history of loss, natural loss, or traumatic loss) using χ^2^ tests for categorical variables and analysis of variance or *t* tests for continuous variables. Next, we calculated the prevalence of PGD, PTSD, and MDD based on symptom criteria using conservative cutoff scores as well as duration and impairment criteria for each disorder. Individuals reporting no loss were excluded from the analysis of PGD prevalence. As a final step, 3 binary logistic regression models were constructed to examine the association between demographic and loss-related risk factors and positive screening results for PGD, PTSD, and MDD. Demographic and loss-related risk factors were selected for inclusion based on the extant literature^8,24,25,26^ and to account for differences across study subgroups. The significance threshold was set at *P* < .05 (2-sided) for all tests. Data were checked for missingness and outliers. All analytic procedures were conducted using SPSS, version 25 (IBM SPSS Inc). Data analysis was conducted between March and June 2023.

## Results

This study included 2034 adults. Their mean (SD) age was 40.7 (15.9) years; 1314 (64.6%) were female and 700 were male (34.4%). Demographic characteristics are displayed in Table 1. Respondents identified as Black (392 [19.3%]), Hispanic (138 [6.8%]), White (1357 [66.7%]), or other race or ethnicity (147 [7.2%]). Most respondents (1394 [68.5%]) had at least some college experience. A total of 505 respondents (24.8%) reported no loss history, 514 (25.3%) reported experiencing a natural loss (eg, due to cancer, fatal cardiac arrest), and 1015 (49.9%) reported experiencing a traumatic loss (eg, homicide, motor vehicle crash, drug-related overdose, or suicide) during their lifetime. Of those who reported experiencing a loss, approximately one-quarter (406 [26.6%]) experienced the death of a nonnuclear family member (eg, cousin or grandparent), followed by the loss of a parent (387 [25.3%]), sibling (245 [16.0%]), close friend (222 [14.5%]), partner or spouse (176 [11.5%]), or child (93 [6.1%]). Respondents reported being very close to the decedent (mean [SD], 8.75 [1.88]), and a majority (809 [52.9%]) reported that the loss had occurred within the previous 5 years.

**Table 1.** Demographic and Loss Characteristics^a^

Characteristic	Total sample (N = 2034)	Loss subgroup			Statistical test result	*P* value
		No history (n = 505)	Natural (n = 514)	Traumatic (n = 1015)		
Age, mean (SD), y	40.7 (15.9)	34.7 (11.4)	49.4 (17.7)	39.5 (14.9)	141.80^b^	<.001
Gender identity						
Female	1314 (64.6)	258 (51.1)	377 (73.3)	679 (66.9)	63.66^c^	<.001
Male	700 (34.4)	236 (46.7)	134 (26.1)	329 (32.4)		
Transgender or nonbinary	21 (1.0)	11 (2.2)	3 (0.6)	7 (0.5)		
Race and ethnicity						
Black	392 (19.3)	92 (18.2)	145 (28.2)	155 (15.3)	66.01^c^	<.001
Hispanic	138 (6.8)	40 (7.9)	17 (3.3)	81 (8.0)		
White	1357 (66.7)	314 (62.2)	326 (63.4)	717 (70.6)		
Other^d^	147 (7.2)	59 (11.7)	26 (5.1)	62 (6.1)		
Education						
Some high school	92 (4.5)	27 (5.3)	16 (3.1)	49 (4.8)	39.19^c^	<.001
High school graduate	549 (27.0)	136 (26.9)	136 (26.5)	277 (27.3)		
Some college	617 (30.3)	122 (24.2)	158 (30.7)	337 (33.2)		
College graduate	521 (25.6)	125 (24.8)	135 (26.3)	261 (25.7)		
Postgraduate	256 (12.6)	95 (18.8)	69 (13.4)	91 (9.0)		
Household income, $						
<19 000	441 (21.7)	107 (21.2)	104 (20.2)	230 (22.7)	66.88^c^	<.001
20 000-49 999	645 (31.7)	108 (21.3)	187 (36.4)	350 (34.5)		
50 000-99 999	563 (27.6)	139 (27.6)	146 (28.4)	278 (27.4)		
>100 000	385 (18.9)	151 (29.9)	77 (15.0)	157 (15.4)		
Relationship with the decedent						
Partner or spouse	NA	NA	43 (8.4)	133 (13.1)	239.59^c^	<.001
Child	NA	NA	16 (3.1)	77 (7.6)		
Parent	NA	NA	232 (45.1)	155 (15.3)		
Sibling	NA	NA	36 (7.0)	209 (20.6)		
Nonnuclear family member^e^	NA	NA	164 (31.9)	242 (23.8)		
Close friend	NA	NA	23 (4.5)	199 (19.6)		
Predeath closeness with the decedent, mean (SD)^f^	NA	NA	9.16 (1.6)	8.54 (2.0)	6.12^g^	<.001
Time since loss, y						
<1	NA	NA	87 (16.9)	239 (23.5)	12.46^c^	.006
1-5	NA	NA	157 (30.5)	326 (32.1)		
6-10	NA	NA	109 (21.2)	191 (18.8)		
>10	NA	NA	161 (31.3)	259 (25.5)		

Abbreviation: NA, not applicable.

^a^ Unless indicated otherwise, values are presented as No. (%) of respondents.

^b^ *F* test.

^c^ χ^2^ Test.

^d^ Includes American Indian or Alaska Native, Asian, Hawaiian or Other Pacific Islander, or multiple races or ethnicities.

^e^ Includes aunt, uncle, cousin, or grandparent.

^f^ Reported on a scale from 1 to 10.

^g^ *t* Test.

### Prevalence of Presumptive PGD, PTSD, and MDD

Prevalence rates of PGD, PTSD, and MDD are presented in Table 2. Overall, most survey respondents did not have a positive screening result for a presumptive mental health diagnosis (1212 [59.6%]). Among the bereaved respondents, 312 (20.4%) had a positive screening result for PGD, with higher rates reported among those who experienced a traumatic loss (241 [23.7%]) (χ^2^_1_ = 20.72; *P* < .001). Nearly one-third of the total sample had a positive screening result for PTSD (633 [31.1%]), including one-third of bereaved adults (518 [33.9%]), with higher rates among those reporting a traumatic loss (409 [40.3%]) (χ^2^_2_ = 79.85; *P* < .001). More than one-quarter of the sample (569 [28.0%]) had a positive screening result for MDD, including nearly one-third of bereaved adults (461 [30.2%]), with a larger proportion in the traumatic loss group (364 [35.9%]) (χ^2^_2_ = 63.36; *P* < .001).

**Table 2.** Mental Health Prevalence Among All Respondents^a^

Mental health disorder	Full sample (N = 2034)	Loss subgroup		
		No history (n = 505)	Natural (n = 514)	Traumatic (n = 1015)
0	1212 (59.6 [57.4-61.7])	353 (69.9 [65.9-73.9])	373 (72.6 [68.7-76.4])	486 (47.9 [44.8-50.9])
≥1				
PTSD	633 (31.1 [29.1-33.2])	115 (22.8 [19.1-26.4])	109 (21.2 [17.7-24.8])	409 (40.3 [37.3-43.4])
PGD	312 (20.4 [18.4-22.5])	NA	71 (13.8 [10.8-16.8])	241 (23.7 [21.2-26.5])
MDD	569 (28.0 [26.0-29.9])	108 (21.4 [17.8-24.9])	97 (18.9 [15.5-22.3])	364 (35.9 [32.9-38.9])
≥2	512 (25.2 [23.3-27.1])	71 (14.1 [11.0-17.1])	96 (18.7 [15.3-22.1])	345 (34.0 [31.1-36.9])

Abbreviations: MDD, major depressive disorder; NA, not applicable; PGD, prolonged grief disorder; PTSD, posttraumatic stress disorder.

^a^ Data are presented as No. (% [95% CI]) of respondents.

### Prevalence of Comorbid Conditions

As shown in Table 2, greater than one-quarter of bereaved adults met presumptive criteria for at least 2 mental health conditions (441 [28.8%]). Table 3 presents single morbidity and comorbidity prevalence rates among subgroups. Among respondents who had a positive screening result for at least 1 disorder, the most common occurrence was a positive screening result for 2 or more concurrent mental health conditions (512 [25.2%]). Participants reporting no loss history were nearly twice as likely to have a positive screening result for both PTSD and MDD (71 [14.1%]) than for PTSD (44 [8.7%]) or MDD (37 [7.3%]) alone. The Figure illustrates the relative proportional rates of PGD, PTSD, and MDD among the natural and traumatic loss samples. Bereaved respondents were more likely to have a positive screening result for PGD, PTSD, and MDD (180 [11.8%]) (Table 3) than any 2 co-occurring or isolated conditions; furthermore, the prevalence of co-occurring PGD, PTSD, and MDD was nearly twice the rate among those reporting a traumatic loss (140 [13.8%]) compared with those reporting a natural loss (40 [7.8%]).

**Table 3.** Presumptive Mental Health Disorders and Comorbidities Among Study Subgroups^a^

Mental health disorder	Loss subgroup		
	No history (n = 505)	Natural (n = 514)	Traumatic (n = 1015)
PGD only	NA	6 (1.2 [0.2-2.1])	20 (2.0 [1.1-2.8])
PTSD only	44 (8.7 [6.2-11.2])	11 (2.1 [0.9-3.4])	52 (5.1 [3.8-6.5])
MDD only	37 (7.3 [5.1-9.6])	18 (3.5 [1.9-5.1])	84 (8.3 [6.6-9.9])
PGD + PTSD	NA	17 (3.3 [1.8-4.9])	65 (6.4 [4.9-7.9])
PGD + MDD	NA	8 (1.6 [0.4-2.6])	16 (1.6 [0.8-2.3])
PTSD + MDD	71 (14.1 [11.0-17.1])	31 (6.0 [4.0-8.1])	124 (12.2 [10.2-14.2])
PGD + PTSD + MDD	NA	40 (7.8 [5.5-10.1])	140 (13.8 [11.7-15.9])

Abbreviations: MDD, major depressive disorder; NA, not applicable; PGD, prolonged grief disorder; PTSD, posttraumatic stress disorder.

^a^ Data are presented as No. (% [95% CI]) of respondents.

**Figure.**
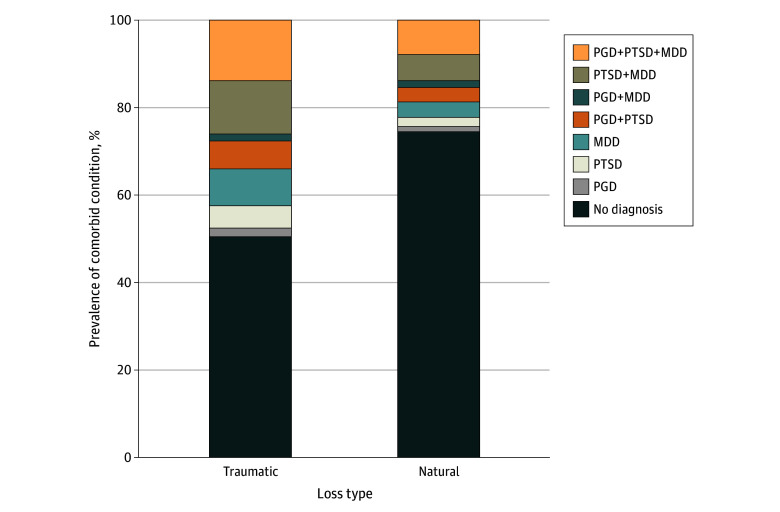
Prevalence of Prolonged Grief Disorder (PGD), Posttraumatic Stress Disorder (PTSD), Major Depressive Disorder (MDD), and Comorbid Conditions Among Natural and Traumatic Loss Subgroups

### Risk Factors for PGD, PTSD, and MDD

Follow-up tests were conducted to investigate the association of sociodemographic and loss-related factors with PGD, PTSD, and MDD among bereaved respondents (Table 4). With respect to sociodemographic variables, age was significantly related to presumptive mental health diagnoses; increased age was associated with a lower risk of PGD, PTSD, or MDD (OR, 0.96 [95% CI, 0.95-0.97] for all 3). Education level was associated with a presumptive PTSD diagnosis (β = −0.51 to −0.81; Wald = 13.52; *P* = .01) but not MDD (Wald = 8.03; *P* = .09) or PGD (Wald = 9.02; *P* = .06). In the case of PTSD, individuals with a high school degree, some college education, or both were less likely than individuals with a postgraduate degree to meet criteria for PTSD, whereas individuals with less than a high school education were just as likely to meet criteria. Greater household income was associated with greater protection from PTSD (Wald = 7.73; *P* = .03) and MDD (Wald = 9.66; *P* = .02) but not PGD (Wald = 2.34; *P* = .14); individuals reporting a household income of $50 000 or greater were significantly less likely to meet criteria for PTSD, and individuals reporting income between $50 000 and $99 999 were significantly less likely to meet criteria for MDD compared with those with a household income of less than $19 000.

**Table 4.** Factors Associated With Presumptive PGD, PTSD, and MDD Among Bereaved Adults

Factor	Mental health disorder among bereaved respondents (n = 1529)								
	PGD			PTSD			MDD		
	β (SE)	*P* value	OR (95% CI)	β (SE)	*P* value	OR (95% CI)	β (SE)	*P* value	OR (95% CI)
Age	−0.04 (0.01)	<.001	0.96 (0.95-0.97)	−0.04 (0.01)	<.001	0.96 (0.95-0.97)	−0.04 (0.01)	<.001	0.96 (0.95-0.97)
Gender identity^a^									
Female	−0.29 (0.17)	.08	0.75 (0.54-1.04)	−0.17 (0.14)	.23	0.85 (0.65-1.11)	−0.11 (0.14)	.41	0.89 (0.68-1.17)
Male	NA	NA	1 [Reference]	NA	NA	1 [Reference]	NA	NA	1 [Reference]
Race and ethnicity									
Black	−0.12 (0.20)	.56	0.89 (0.60-1.32)	−0.38 (0.17)	.02	0.68 (0.49-0.95)	−0.01 (0.17)	.97	0.99 (0.72-1.37)
Hispanic	0.01 (0.28)	.97	0.99 (0.56-1.74)	−0.08 (0.24)	.74	0.92 (0.58-1.48)	−0.12 (0.24)	.61	0.88 (0.55-1.42)
White	NA	NA	1 [Reference]	NA	NA	1 [Reference]	NA	NA	1 [Reference]
Other^b^	0.23 (0.31)	.46	1.26 (0.69-2.29)	0.13 (0.25)	.62	1.13 (0.69-1.86)	−0.31 (0.27)	.25	0.74 (0.43-1.25)
Education									
Some high school	−0.36 (0.44)	.41	0.70 (0.30-1.64)	−0.51 (0.36)	.16	0.60 (0.29-1.22)	−0.88 (0.38)	.02	0.42 (0.20-0.87)
High school	−0.25 (0.29)	.39	0.78 (0.44-1.38)	−0.81 (0.24)	<.001	0.45 (0.28-0.72)	−0.61 (0.24)	.01	0.54 (0.34-0.88)
Some college	−0.39 (0.28)	.16	0.68 (0.39-1.16)	−0.73 (0.23)	.001	0.48 (0.31-0.75)	−0.39 (0.23)	.09	0.68 (0.43-1.07)
College graduate	−0.64 (0.28)	.02	0.53 (0.30-0.91)	−0.69 (0.23)	.002	0.50 (0.32-0.78)	−0.47 (0.23)	.04	0.62 (0.40-0.97)
Postgraduate degree	NA	NA	1 [Reference]	NA	NA	1 [Reference]	NA	NA	1 [Reference]
Household income, $									
<19 000	NA	NA	1 [Reference]	NA	NA	1 [Reference]	NA	NA	1 [Reference]
20 000-49 999	0.01 (0.19)	.98	1.01 (0.69-1.47)	−0.25 (0.16)	.13	0.78 (0.56-1.07)	0.03 (0.17)	.85	1.03 (0.74-1.43)
50 000-99 999	−0.14 (0.22)	.51	0.87 (0.57-1.33)	−0.43 (0.18)	.02	0.65 (0.46-0.93)	−0.38 (0.19)	.04	0.68 (0.48-0.98)
>100 000	0.10 (0.29)	.73	1.11 (0.62-1.96)	−0.64 (0.23)	.006	0.53 (0.33-0.83)	0.06 (0.23)	.80	1.06 (0.68-1.66)
Predeath closeness with the decedent	0.21 (0.05)	<.001	1.23 (1.13-1.35)	0.24 (0.04)	<.001	1.27 (1.18-1.36)	0.11 (0.03)	.001	1.12 (1.05-1.19)
Partner or spouse	1.31 (0.30)	<.001	3.69 (2.04-6.69)	0.75 (0.24)	.002	2.13 (1.32-3.39)	0.75 (0.24)	.002	2.11 (1.31-3.41)
Child	1.75 (0.34)	<.001	5.73 (2.93-11.18)	1.42 (0.29)	<.001	4.14 (2.34-7.32)	1.01 (0.30)	<.001	2.75 (1.54-4.92)
Parent	1.01 (0.27)	<.001	2.72 (1.60-4.62)	0.55 (0.21)	.01	1.74 (1.14-2.64)	0.61 (0.22)	.005	1.83 (1.19-2.81)
Sibling	0.73 (0.28)	.008	2.07 (1.21-3.56)	0.46 (0.22)	.03	1.59 (1.04-2.44)	0.39 (0.22)	.08	1.47 (0.95-2.23)
Nonnuclear family member^c^	0.20 (0.27)	.47	1.22 (0.72-2.06)	0.18 (0.20)	.37	1.19 (0.81-1.76)	0.35 (0.20)	.08	1.41 (0.96-2.09)
Friend	NA	NA	1 [Reference]	NA	NA	1 [Reference]	NA	NA	1 [Reference]
Time since loss, y									
<1	NA	NA	NA	0.78 (0.20)	<.001	2.19 (1.48-3.23)	0.83 (0.20)	<.001	2.29 (1.54-3.38)
1-5	0.77 (0.20)	<.001	2.17 (1.48-3.18)	0.58 (0.18)	.001	1.78 (1.26-2.53)	0.44 (0.18)	.02	1.55 (1.08-2.21)
6-10	0.60 (0.21)	.004	1.82 (1.21-2.74)	0.55 (0.19)	.004	1.74 (1.19-2.53)	0.23 (0.20)	.25	1.26 (0.85-1.86)
>10	NA	NA	1 [Reference]	NA	NA	1 [Reference]	NA	NA	1 [Reference]
Cause of death									
Traumatic loss	0.61 (0.19)	<.001	1.84 (1.28-2.64)	0.71 (0.15)	<.001	2.03 (1.50-2.74)	0.71 (0.16)	<.001	2.03 (1.49-2.76)
Natural loss	NA	NA	1 [Reference]	NA	NA	1 [Reference]	NA	NA	1 [Reference]

Abbreviations: MDD, major depressive disorder; NA, not applicable; OR, odds ratio; PGD, prolonged grief disorder; PTSD, posttraumatic stress disorder.

^a^ Individuals who identified as gender nonconforming were excluded due to a lack of power.

^b^ Includes American Indian or Alaska Native, Asian, Hawaiian or Other Pacific Islander, or multiple races or ethnicities.

^c^ Includes aunt, uncle, cousin, or grandparent.

In terms of loss-related characteristics, bereaved individuals reporting greater psychological closeness with the decedent were more likely to meet criteria for a presumptive diagnosis of PGD (Wald = 16.08; *P* < .001), PTSD (Wald = 40.61; *P* < .001), and MDD (Wald = 10.50; *P* < .01). Moreover, those with less time elapsed since the death (ie, 1-5 years post loss) were approximately twice as likely to meet criteria for PGD (Wald = 15.58; *P* < .001) and PTSD (Wald = 10.46; *P* = .001), and they were 1.5 times as likely to meet criteria for MDD relative to those who experienced a loss over 10 years prior (Wald = 5.75; *P* = .01). Similarly, relationship with the decedent was related to PGD, PTSD, and MDD (Wald range, 15.55-44.37; all *P* < .001), with the loss of a child, followed by the loss of a partner or spouse, presenting the greatest level of risk across all presumptive diagnoses. The decedent’s cause of death was also associated with presumptive mental health morbidity, even after accounting for demographic and other loss-related characteristics. Specifically, individuals reporting a traumatic loss were more likely to meet presumptive criteria for PGD (Wald = 10.71; *P* = .001), PTSD (Wald = 21.32; *P* < .001), and MDD (Wald = 20.22; *P* < .001) than those reporting natural losses.

## Discussion

In this study with a cross-sectional convenience sample, psychiatric morbidity among bereaved US adults was common, and especially high rates of morbidity were observed among adults with a history of losses due to potentially traumatic causes of death (eg, homicide, motor vehicle crash, drug-related overdose, or suicide). Compared with bereaved individuals with a history of losses due to natural causes, those with a history of traumatic losses were twice as likely to have a positive screening result for PGD, PTSD, and MDD. Moreover, the proportion of individuals with a positive screening result for multiple conditions was nearly twice as high in the traumatic loss group compared with the natural and no-loss groups. This finding is especially interesting given that more than one-quarter of the deaths (406 [26.6%]) were among nonnuclear family members.

These findings extend research from several smaller studies with subpopulations of traumatic loss survivors,^27,28,29^ suggesting that a traumatic loss may place bereaved family members and friends of the decedent at especially heightened risk of psychiatric complications like PGD, PTSD, and MDD.^30,31^ However, a confounding issue is that these deaths disproportionately affect young individuals, often violating social and developmental expectations around the natural life course. Fatal accidents and injuries, suicide, and homicide account for the 3 leading causes of death among individuals aged younger than 44 years in the US.^32^ Compared with individuals grieving natural losses, those with a history of traumatic losses in our sample were more likely to have survived the death of a child, partner or spouse, or sibling. In our multivariate analyses, respondents grieving the death of a child were nearly 3 to 5 times as likely to have a positive screening result for PGD, PTSD, or MDD or their co-occurrence compared with those grieving the death of a close friend.

In this study, socioeconomic and racial and ethnic disparities in bereavement from all forms of dying were observed that were further associated with the risk of bereavement-related psychiatric morbidity. Individuals in the no-loss group were more likely to be male, to have a postgraduate degree, and to have an annual household income of more than $100 000. In contrast, women, Black respondents, and individuals with an annual household income of less than $50 000 were disproportionately represented among the natural loss group. This finding is consistent with research showing that by age 30 years, Black individuals in the US are twice as likely as White individuals to have lost 2 or more family members.^33^ Epidemiological research has consistently documented an association between socioeconomic inequalities in education and income and life expectancy,^34^ and our results suggest that these inequalities may similarly contribute to the likelihood of experiencing the death of a loved one and psychiatric complications due to bereavement. Education was associated with a greater risk of PTSD, and income was associated with a greater risk of both PTSD and MDD, in which individuals in the highest income brackets had a decreased risk of both conditions compared with individuals in the lowest income bracket. In a more nuanced look at the findings related to education and PTSD, respondents with a high school degree or less and a postgraduate degree had a similar risk of PTSD, whereas those with at least some college had a lower risk of PTSD. Although lower education can increase the risk of PTSD,^35^ the protective benefits of education are diminished at the highest levels of education,^36^ possibly due to overeducation (eg, job-education mismatch), student loan burdens, or other factors, which may explain these findings.

### Limitations

This study has several limitations. Its cross-sectional design precluded our ability to make causal inferences about factors associated with mental health outcomes. The study design also limited our ability to determine whether reported mental health symptoms may have, in some cases, preceded or were the direct result of the loved one’s death, although it is likely that bereavement would exacerbate any preexisting symptoms. Prevalence estimates of PGD, PTSD, and MDD were derived from self-report measures, which may inflate estimates compared with clinical interviews.^37^ Future studies using structured clinical interviews are needed to confirm these rates in other US samples. Relatedly, this study used a single item, rather than a validated instrument, to assess predeath closeness with the decedent. The use of Qualtrics panels of respondents motivated by some financial incentive to participate potentially affects the generalizability of the results. Finally, historical factors, such as the recent COVID-19 pandemic, may have contributed to the high rate of reported mental health outcomes.^38^

## Conclusions

This survey study characterizes the risk of adverse mental health consequences, including PGD, PTSD, and MDD, among bereaved adults in the US, particularly among those who have experienced a traumatic loss. Given the substantial levels of comorbidities observed, these findings underscore the need for integrated psychological care that leverages transdiagnostic mechanisms of evidence-based practice.

## Notes

Supplement 1. **eFigure.** Recruitment and Sampling Procedures Flowchart

Supplement 2.
Data Sharing Statement
