# Supplementary material for: Prevalence and Co-Occurrence of Psychiatric Conditions Among Bereaved Adults
Source: JAMA Netw Open. 2024 Jun 6;7(6):e2415325. doi: 10.1001/jamanetworkopen.2024.15325 (PMC11157353; doi:10.1001/jamanetworkopen.2024.15325)
Supplement: Supplement 1. — eFigure. Recruitment and Sampling Procedures Flowchart [file jamanetwopen-e2415325-s001.pdf]

Supplementary Online Content

Rheingold AA, Williams JL, Bottomley JS. Prevalence and co-occurrence of psychiatric conditions among bereaved adults. *JAMA Netw Open*. 2024;7(6):e2415325.
doi:10.1001/jamanetworkopen.2024.15325

eFigure. Recruitment and Sampling Procedures Flowchart

This supplementary material has been provided by the authors to give readers additional information about their work.

© 2024 Rheingold AA et al. *JAMA Network Open.*

eFigure. Recruitment and Sampling Procedures Flowchart

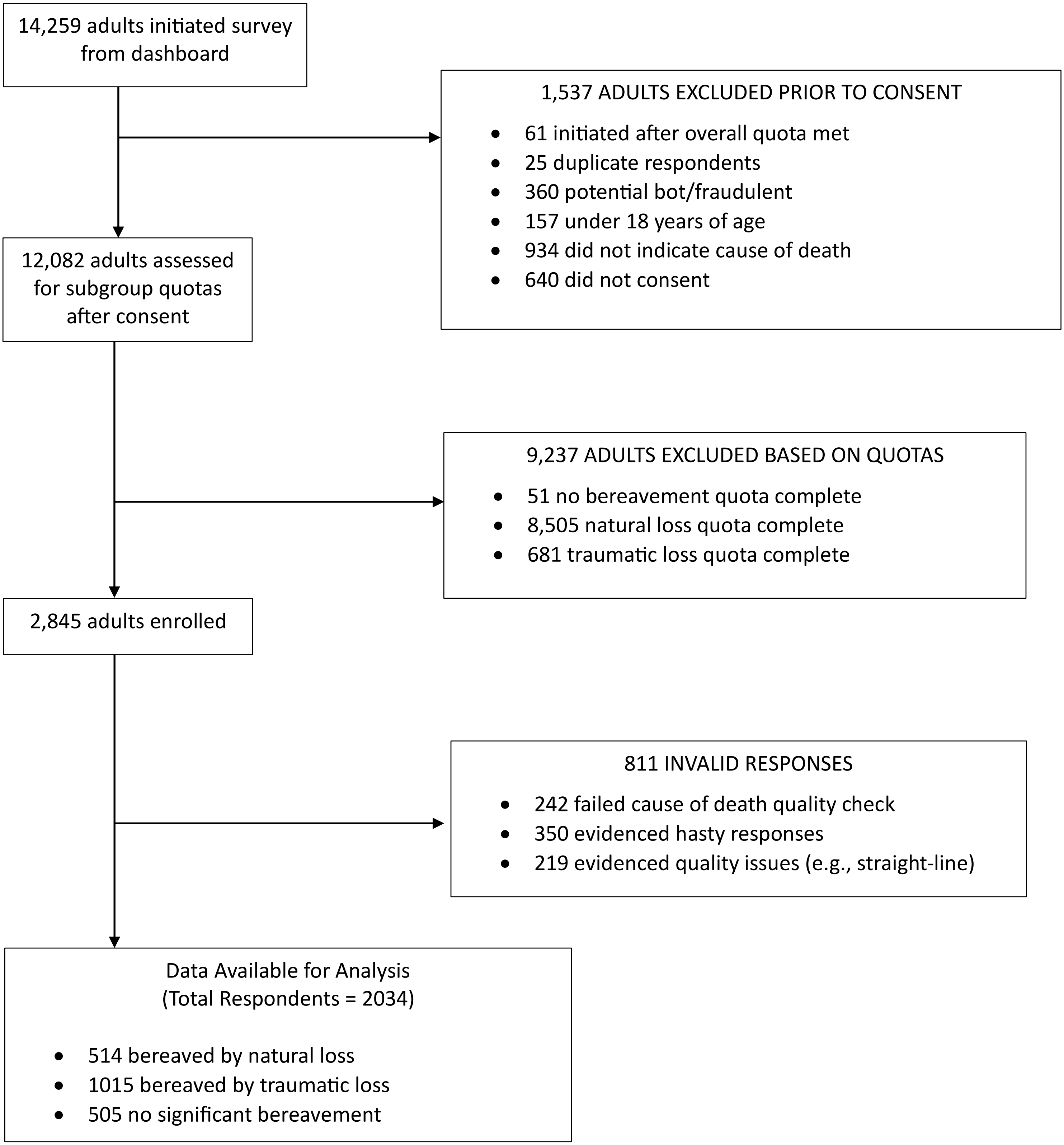

© 2024 Rheingold AA et al. *JAMA Network Open.*
