# Supplementary material for: Prevalence and Co-Occurrence of Psychiatric Conditions Among Bereaved Adults
Source: JAMA Netw Open. 2024 Jun 6;7(6):e2415325. doi: 10.1001/jamanetworkopen.2024.15325 (PMC11157353; doi:10.1001/jamanetworkopen.2024.15325)
Supplement: Supplement 2. — Data Sharing Statement [file jamanetwopen-e2415325-s002.pdf]

Data Sharing Statement

Rheingold. Prevalence and Co-Occurrence of Psychiatric Conditions Among Bereaved Adults. *JAMA Netw Open*. Published June 06, 2024. doi:10.1001/jamanetworkopen.2024.15325

Data
Data available: Yes
Data types: Deidentified participant data, Data dictionary
How to access data: The data that support the findings of this study are available from the corresponding author, Alyssa Rheingold at rheingaa@musc.edu, upon reasonable request.
When available: With publication

Supporting Documents
Document types: None

Additional Information
Who can access the data: Researchers whose proposed use of the data has been approved.
Types of analyses: for any purpose
Mechanisms of data availability: With a signed data access agreement.
